# Sensitivity, specificity, and accuracy of a liquid biopsy approach utilizing molecular amplification pools

**DOI:** 10.1038/s41598-021-89592-8

**Published:** 2021-05-24

**Authors:** Jessica Garcia, Nick Kamps-Hughes, Florence Geiguer, Sébastien Couraud, Brice Sarver, Léa Payen, Cristian Ionescu-Zanetti

**Affiliations:** 1 Laboratoire de Biochimie Et Biologie Moléculaire, Groupe Hospitalier Sud, Hospices Civils de Lyon, 69495 Pierre Bénite, France; 2 CIRculating CANcer (CIRCAN) Program, Hospices Civils de Lyon Cancer Institute, 69495 Pierre Bénite, France; 3 Fluxion Biosciences, Alameda, CA USA; 4 CIRculating CANcer (CIRCAN) Program, Acute Respiratory Disease and Thoracic Oncology Department, Lyon Sud Hospital, Cancer Institute of Hospices Civils de Lyon, Lyon, France

**Keywords:** Cancer, Computational biology and bioinformatics

## Abstract

Circulating cell-free DNA (cfDNA) has the potential to be a specific biomarker for the therapeutic management of lung cancer patients. Here, a new sequencing error-reduction method based on molecular amplification pools (MAPs) was utilized to analyze cfDNA in lung cancer patients. We determined the accuracy of MAPs plasma sequencing with respect to droplet digital polymerase chain reaction assays (ddPCR), and tested whether actionable mutation discovery is improved by next-generation sequencing (NGS) in a clinical setting. This study reports data from 356 lung cancer patients receiving plasma testing as part of routine clinical management. Sequencing of cfDNA via MAPs had a sensitivity of 98.5% and specificity 98.9%. The ddPCR assay was used as the reference, since it is an established, accurate assay that can be performed contemporaneously on the same plasma sample. MAPs sequencing detected somatic variants in 261 of 356 samples (73%). Non-actionable clonal hematopoiesis-associated variants were identified via sequencing in 21% of samples. The accuracy of this cfDNA sequencing approach was similar to that of ddPCR assays in a clinical setting, down to an allele frequency of 0.1%. Due to broader coverage and high sensitivity for insertions and deletions, sequencing via MAPs afforded important detection of additional actionable mutations.

## Introduction

A growing understanding of cancer molecular complexity and the role of oncogenic drivers such as mutations in genes encoding the epidermal growth factor receptor (*EGFR*), V-Ki-Ras2 Kirsten Rat Sarcoma 2 (*KRAS*), (MET) and Anaplastic Lymphoma Kinase (ALK) genes have ushered in the era of targeted therapies. Molecular profiling of a cancer patient’s tumor to reveal targetable alterations is an important first step in the personalization of cancer treatment plans. Usually, these molecular mutational analyses are performed on formalin-fixed paraffin-embedded (FFPE) tissue samples at diagnosis or recurrence.

Biopsies of advanced stage non-small cell lung cancer (NSCLC) need invasive exams in fragile patients, and therefore minimally invasive “liquid biopsies” have generated considerable enthusiasm. In fact, the use of cfDNA for sensitizing and resistance somatic mutation detection in oncodrivers for NSCLC was integrated into the European Medicines Agency (EMA) approval. It is now possible to detect somatic alterations from minute cfDNA concentrations (less than 0.1%), enabling the detection of alterations in low-volume plasma samples. Several techniques are currently available to detect cfDNA, including highly sensitive PCR (polymerase chain reaction) assays and next-generation sequencing (NGS). In previous work, we highlighted the complementarity between the RAS droplet digital polymerase chain reaction (ddPCR) method and NGS, which often confirmed ddPCR results and provided a larger overview of the major targetable alterations of 56 genes (56G oncology panel, Swift Biosciences, Ann Arbor, MI, USA) in one run at diagnosis with a 0.5% threshold in lung and colon cancers^1^.

Sequencing-based liquid biopsy testing offers tremendous promise for tailoring treatment regimens to the changing tumor genomic landscape. Current applications include replacing tissue testing when that is difficult or fails, re-testing as disease progresses on treatment, and earlier detection of drug resistance. Despite the successful commercial introduction of liquid biopsy testing in oncology^2–4^, mutation detection performance limitations remain^5–7^. A better understanding of test performance and clinical utility and higher-accuracy methods are needed to broaden adoption of liquid biopsies as the standard of care in cancer treatment.

The accuracy of liquid biopsy tests is an area of constant improvement and scientific debate. Publications have reported excellent agreement between cfDNA liquid biopsy testing and either tissue or blood-based PCR testing^8–10^, primarily looking at NSCLC and colorectal cancer (CRC) in patients at advanced stage (Stage III-IV). In contrast, studies using more challenging indications and early-stage samples have generated data that demonstrate significant room for improvement for the currently-available clinical assays in this setting^5,7,11^. For example, a comparison of two sequencing lab-developed tests (LDTs) showed low concordance (< 30%) in early-stage patient samples^7^, while another independent study reported low positive concordance of 15% to tissue testing^5^. Researchers have also run 4 commercial liquid biopsy LDTs in parallel on matched blood samples with tissue data, finding low concordance between the different tests, especially in the space below 1% mutant allele frequency^6^. The discrepancy between data sets in challenging sample cohorts highlights the need for further orthogonal testing in the clinical setting and sensitivity/specificity improvements, especially in the 0.1%-1% allelic fraction (AF) range, where most of the false positives and false negatives have been found in orthogonal studies^6^. Current sequencing-based liquid biopsy clinical tests rely on unique molecular identifiers (UMIs) to reduce noise. UMIs are short DNA strands used to tag each starting molecule so that it can be tracked through the amplification and sequencing process. The key challenges for UMIs include low tagging efficiency for limited cfDNA input material, replication errors in the UMIs themselves, and high costs due to the sequencing depths required^12^. For advanced-stage patients, recent work has shown that a large majority of patients responded to targeted therapy prescribed based on a commercial cfDNA sequencing test, demonstrating the clinical utility of such testing^13^. Sequencing and ddPCR can provide complementary information from the same blood sample. With ddPCR, we have a focused view usually associated with higher sensitivity as we demonstrated it in clinical setting for RAS alterations in a colon cancer cohort^1^. Nevertheless, the diversity of the clinical molecular profile of lung cancers and the rarity of cfDNA amounts per sample requires broader oncogene coverage than can be provided by ddPCR.

**Figure 1:**
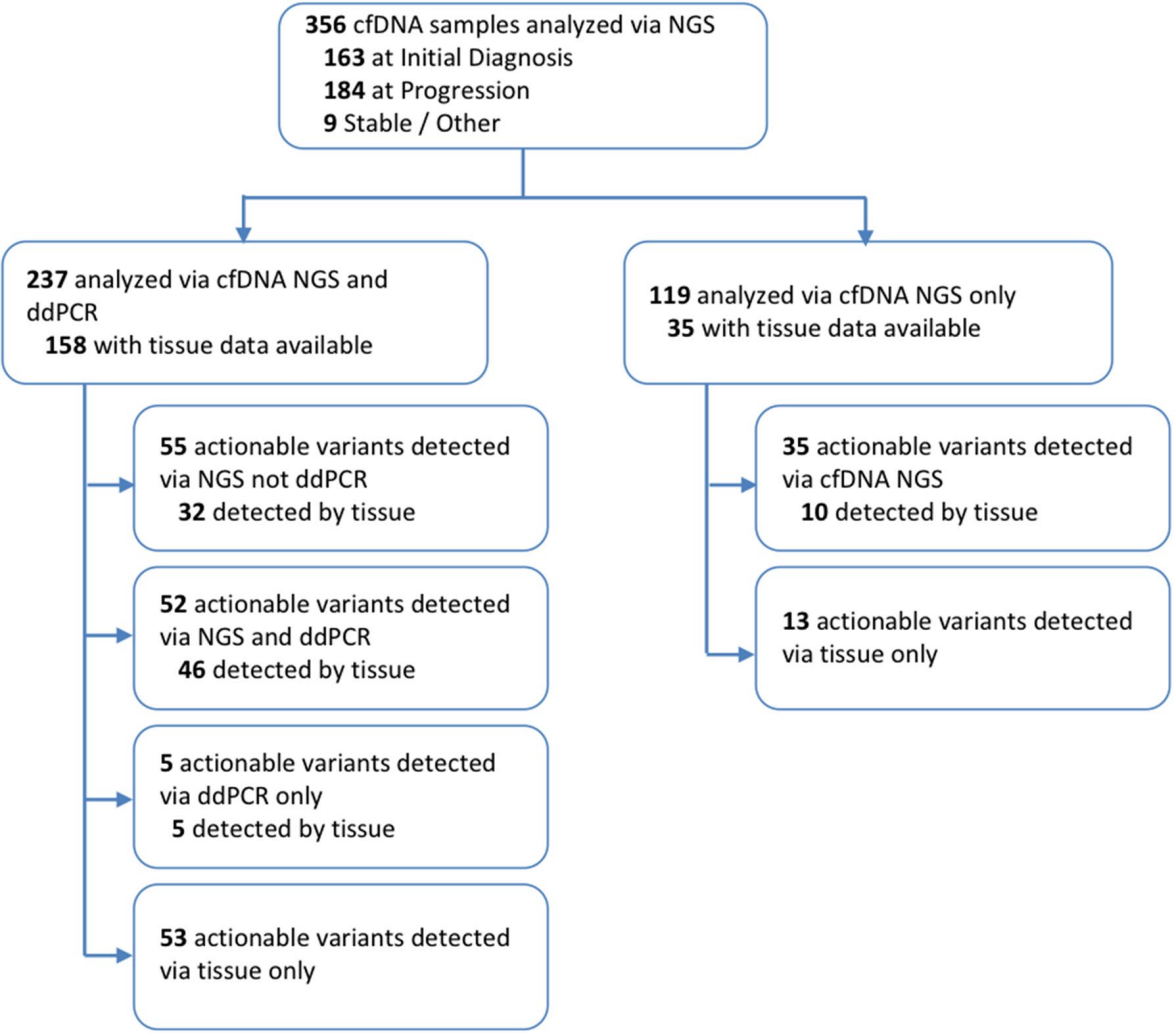
Flow chart summarizing types of tests conducted as part of our study, and numbers of actionable variants detected in each sub-group. 237 patients were analyzed by both cfDNA NGS and ddPCR, and of these tissue NGS data was available for 158 patients. 119 patients were analyzed by cfDNA NGS but not ddPCR; of these, 35 had tissue NGS data available.

Here we present clinical validation for a recently-introduced sequencing error-reduction method based on molecular amplification pools (MAPs) that has shown improved analytical accuracy^14^. This methodology tracks variants present in large collections of molecules, as opposed to single molecule UMIs. Used in conjunction with the ERASE-Seq variant caller (Fluxion Biosciences, Alameda, CA, USA), MAPs provide a widely-applicable approach to ultrasensitive NGS test development. Sensitivity and specificity were measured with respect to ddPCR testing performed as part of standard clinical work-up. This retrospective study reports data from 356 lung cancer patients receiving plasma testing as part of routine clinical management and utilizing the MAPs approach. cfDNA from each sample was separated into two separate pools, and analyzed via NGS on a 56-gene panel as described in the analysis flow chart, Fig. 1. The NGS assay and ddPCR give complementary information for a complete overview of the molecular profile of the patients at all moments of the disease. With ddPCR BEAMing, we have a focused view but usually with higher sensitivity as we demonstrated it in clinical setting for RAS alterations in colon cancer cohort^1^.

The results here present data from a large, orthogonally validated liquid biopsy lung cancer dataset. The advantages in terms of additional actionable variant discovery by NGS as compared to both ddPCR and tissue sequencing were quantified.

## Results

### Somatic variant detection

**Figure 2:**
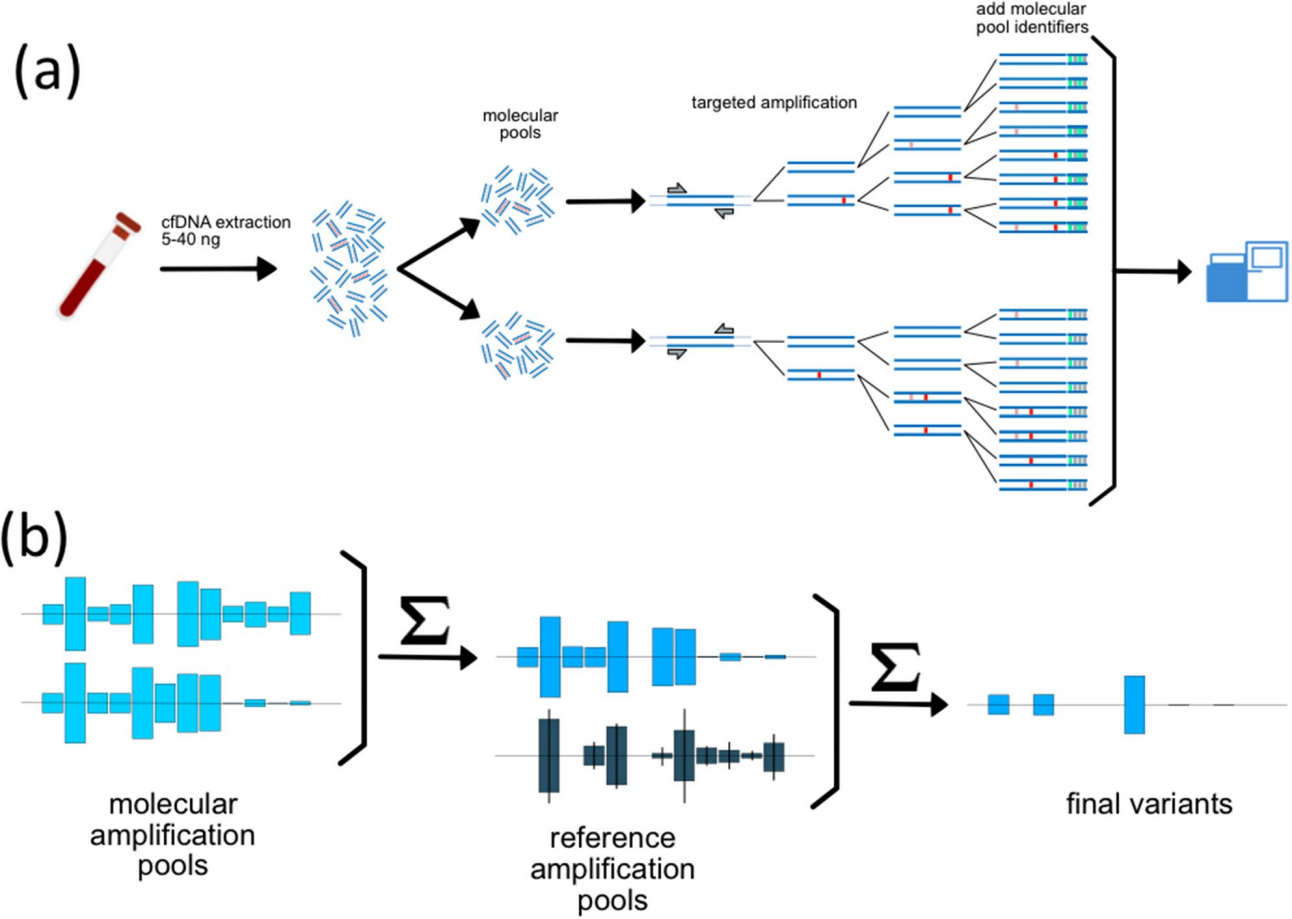
(**a**) Extracted cfDNA is divided into two molecular pools, each containing a fraction of the tumor-derived cfDNA (red fragments). Each molecular pool undergoes a targeted amplification reaction, resulting in the introduction of both stochastic (red) and recurrent (pink) artifacts. Post amplification, each molecular amplification pool (MAP) is indexed and sequenced. (**b**) Variant data from the two molecular pools is statistically compared to eliminate stochastic errors and referenced to a large number of reference molecular pools to eliminate recurrent errors.

**Figure 3:**
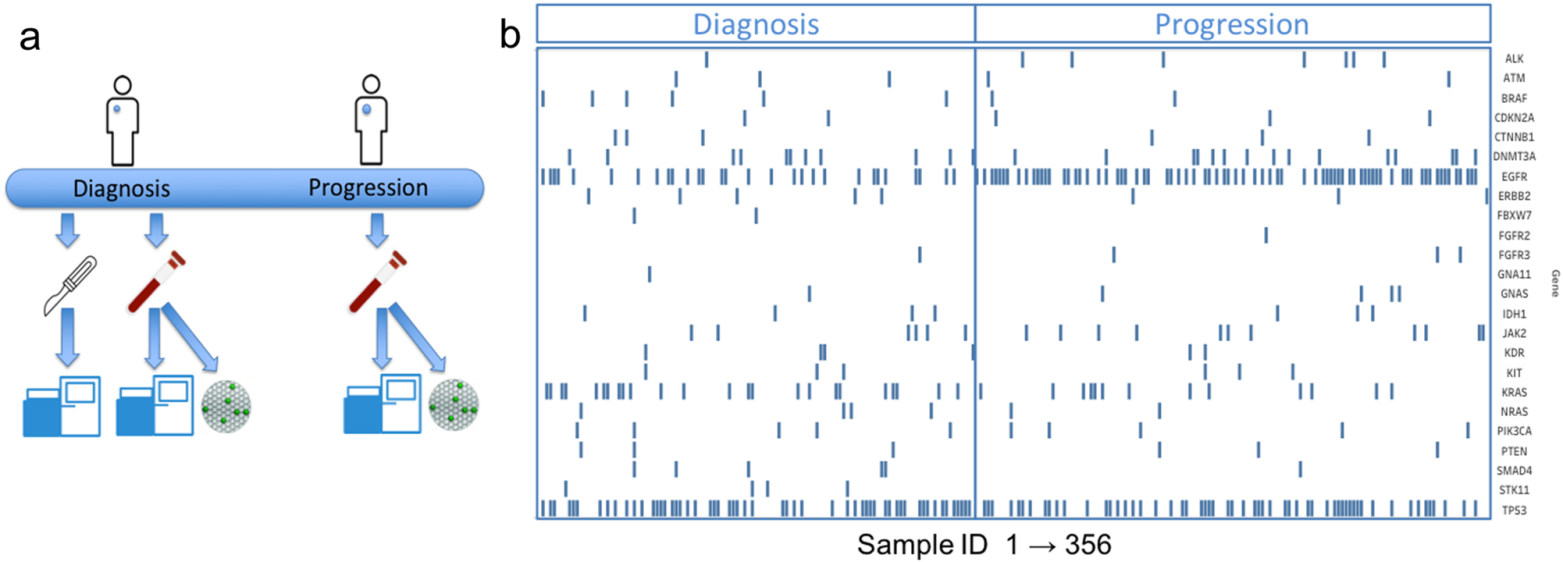
(**a**) Tissue samples were obtained and sequenced at diagnosis. Liquid biopsy blood samples are obtained either at diagnosis or at progression. Resulting cfDNA is sequenced via the MAP method and tested via ddPCR for a clinically-relevant set of variants. (**b**) Gene-level mutational load for all mutation-positive sequencing results at either diagnosis or progression is shown for the 24 most-detected genes as a function of sample number (x-axis). Samples were divided by timepoint: initial diagnosis (left) and progression (right). Samples are not matched and represent the population of mutation-positive samples used in the study that were obtained at each timepoint.

MAP-based mutation detection (Fig. 2) was applied to a set of 356 cfDNA samples obtained in a clinical testing environment and meeting the sequencing quality control metrics for both molecular pools (Fig. 1). The use of a MAP-based confidence score eliminates a majority of false positive calls in the 0.1–1% AF range. Patients were tested at one of two clinical timepoints: initial diagnosis or progression (Fig. 3a). Samples were independently analyzed in Hospices Civils de Lyon’s (HCL) laboratory for ddPCR and NGS and all samples were blinded. The NGS analyses were carried out without knowledge of ddPCR results or clinical information. A matched blood sample was tested via ddPCR (Bio-Rad ddPCR and/or BEAMing) for a set of clinically-actionable variants of the *EGFR* gene: T790M, L858R, and DelEx19. Tissue mutational data was obtained from physician testing records. The sequencing panel contained 263 amplicons across 56 genes covering pan-cancer mutations of literature-cited clinical relevance (Supplementary Table S1). The panel contained all of the variants tested by ddPCR. The ddPCR test method was chosen for comparison based on the fact that it has high sensitivity, covers the EGFR alterations of high clinical utility (Exon 18; 19; 20; 21), and has a low cost in comparison with commercial NGS tests such as Guardant360 (Guardant Health) and FoundationOne Liquid (Foundation Medicine). Additionally, the ddPCR test is an orthogonal method and thus less likely to mask inherent biases that might not be revealed by comparison between NGS-based tests.

**Figure 4:**
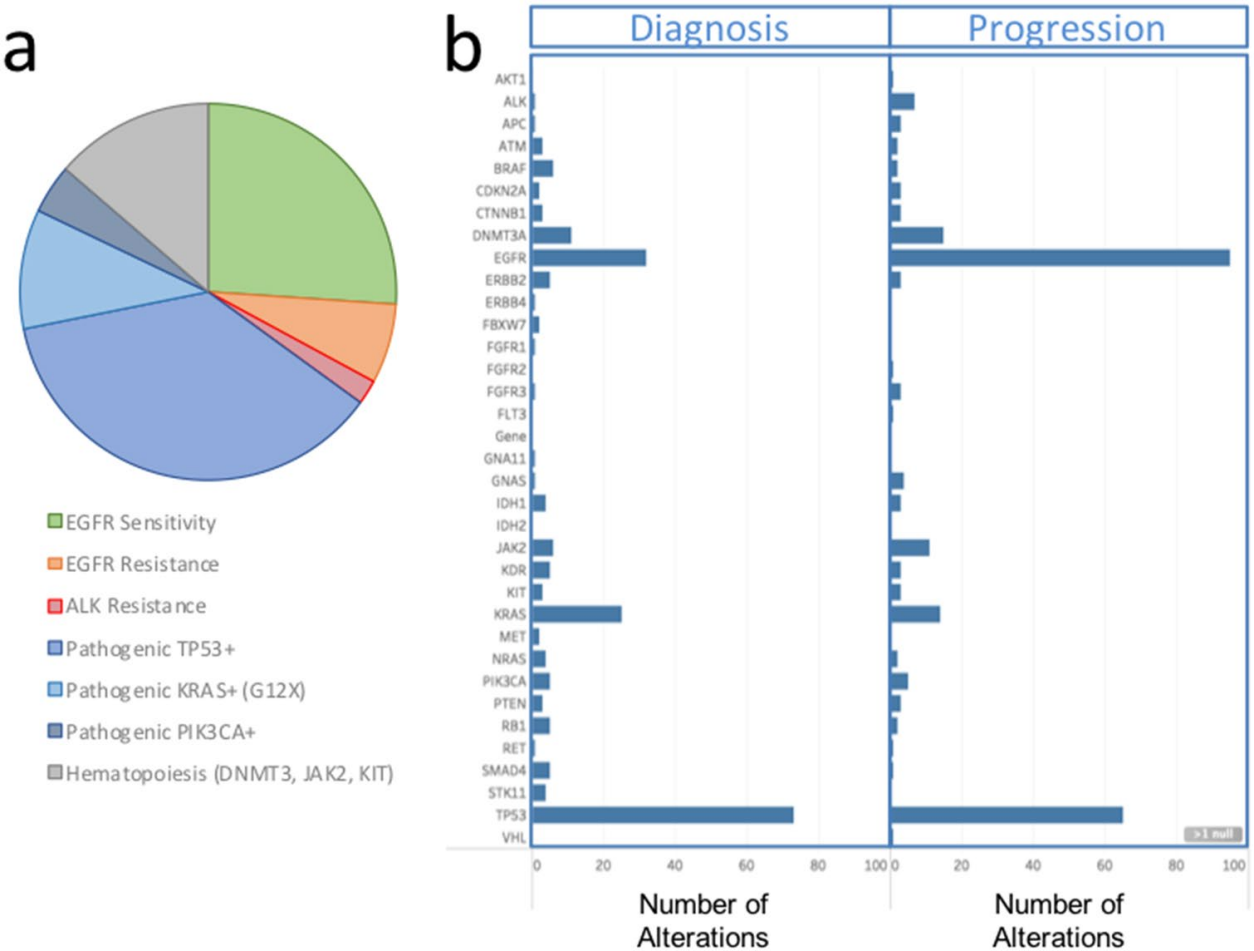
(**a**) The prevalence of clinically-significant mutations over the entire population is presented as a pie chart by type. (**b**) A histogram of gene-level mutational load, with numbers of observations (x-axis) versus gene (y-axis labels). Changes in prevalence between the initial diagnosis cohort (left) and progression cohort (right) are readily observed for the *EGFR* gene.

Gene-level mutational status for all patient samples with at least one mutation detected by liquid biopsy NGS is plotted in Fig. 3b. The full variant set is contained in Supplementary Table S2. Patients’ liquid biopsy samples had an average of 1.7 somatic mutations per sample when sequenced via the MAPs protocol, with a larger number of mutations detected at progression (Fig. 3b). The types of actionable mutations detected included sensitizing/resistance mutations of the *EGFR*, *ALK, BRAF* and *KRAS* mutations (Fig. 3b). Other pathogenic mutations (i.e. *TP53*, *PIK3CA*) were also detected. Clonal hematopoiesis-associated variants (CHIP, clonal hematopoiesis of indeterminate potential) were detected for the *DNMT3*, *JAK2* and *KIT* genes; 27% of *TP53* variants were also found to be CHIP-associated by our definition (Supplementary Table S5 and Supplementary Fig. S1). In agreement with known molecular disease evolution, a high level of alterations in *EGFR* mutation-positive samples was observed at the progression time point as compared to treatment-naïve patients at initial diagnosis. *TP53* mutation rates remained high at both timepoints. The overall prevalence of mutations by type is summarized in Fig. 4.

**Figure 5:**
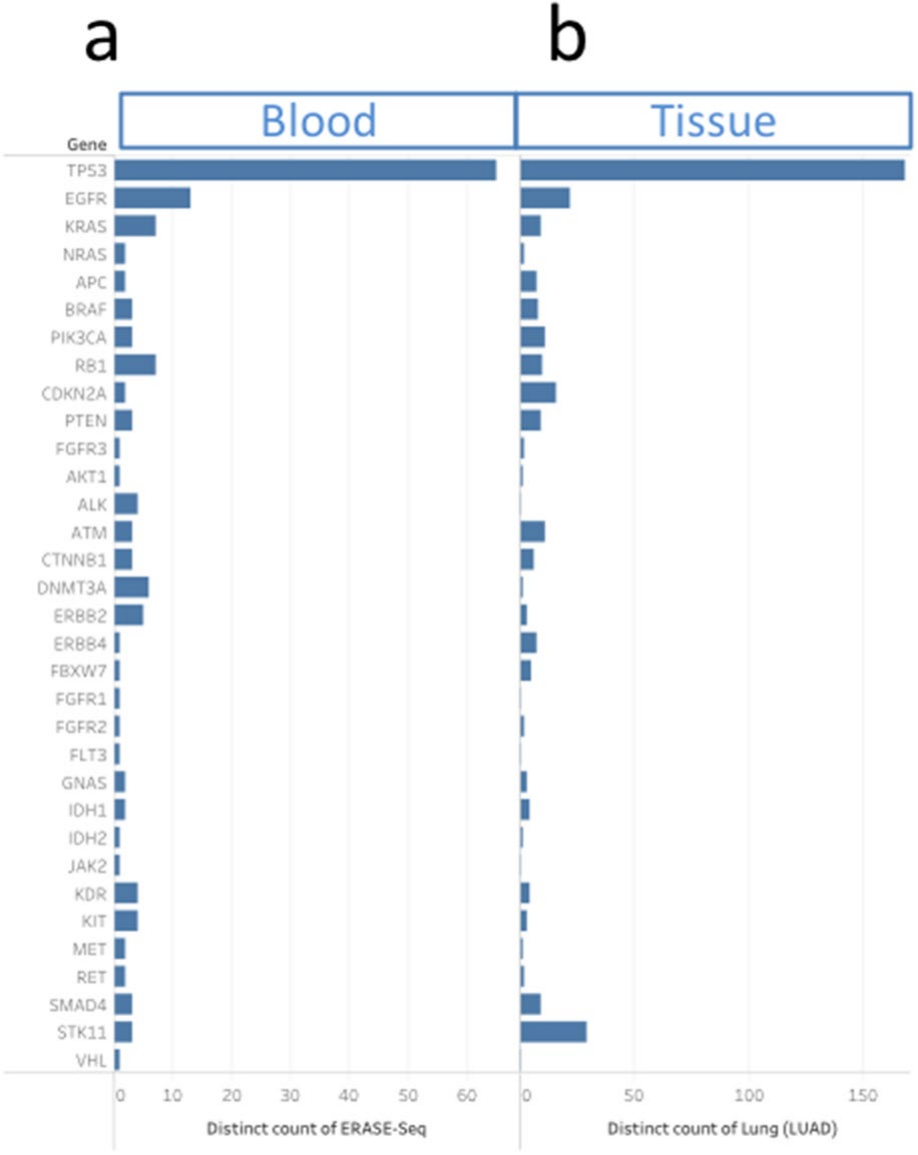
The number of unique mutations detected per gene is plotted for our liquid biopsy data set in lung (**a**) as compared to tissue-based cancer atlas (TCGA) data (**b**). The tissue data set analyzed in the TCGA cancer genome atlas were lung adenocarcinoma. The analysis was restricted to genomic regions covered by both tests, with the highest prevalence genes shown.

The number of unique mutations per gene observed in our 356-sample set is compared to similar measures for lung (Fig. 5) and other cancer tissues (Supplementary Fig. S2) across the same 56-gene pan-cancer targeted regions. The somatic molecular signature of solid tumors in tissue as captured by The Cancer Genome Atlas (TCGA), containing full exon tissue sequencing data from over 300 cancer patients from a number of different indications^15,16^ and crossed with our panel coverage. Agreement with the lung adenocarcinoma data set (R^2^ = 0.91, Fig. 5) is significantly better with respect to unique mutation counts per gene than for melanoma, colon and bladder cancers, demonstrating that the cfDNA assay is accurately capturing lung cancer variant signatures (Supplementary Fig. S2. C, D, E; R^2^ = 0.51, 0.54, 0.86, respectively).

### Blood-to-blood orthogonal concordance

**Figure 6:**
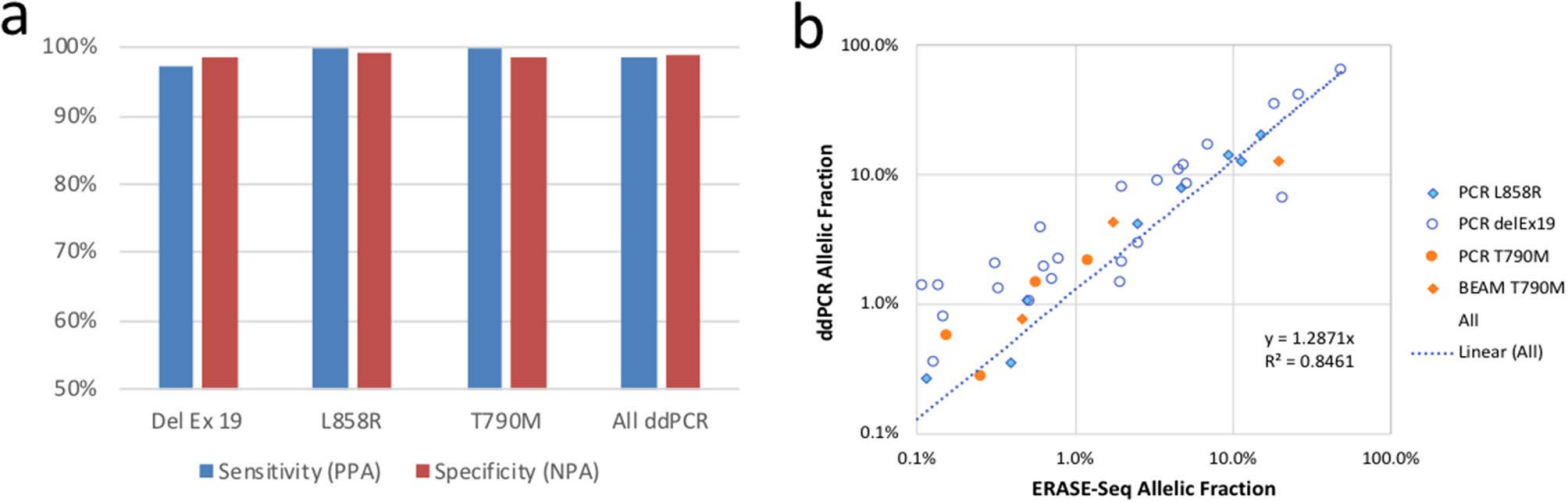
Sensitivity and specificity of sequencing compared to orthogonal ddPCR testing in the blood is presented for three clinically-actionable alterations: exon 19 deletions, L8585R, and T790M mutations (**a**). In addition to each individual biomarker, comprehensive ddPCR sensitivity/specificity for all targets is plotted. A scatter plot compares allele frequencies reported by ddPCR versus ERASE-Seq sequencing results (**b**).

FFPE testing was usually done before treatment, while cfDNA testing was carried out during progression to test for progression mechanisms or a resistant molecular profile. In consequence, the molecular profile of the tumor has been modified with time and treatment, explaining the limited concordance previously observed between cfDNA and tissue biopsies. In addition, the ddPCR assays display high sensitivity as compared to classical NGS tests for focused clinical hot-spots^1^, and are used as routine clinical tests in our hospital at the time of disease progression. Taken together, these constraints supported using ddPCR testing as the reference test for the accuracy comparison. Overall concordance between MAP liquid biopsy data and ddPCR-targeted mutations (Del Ex19, L858R and T790M) was determined above the limits of detection for each variant type (0.1%, 0.1% and 0.13% respectively). The overall concordance across 1016 ddPCR tests was 98.8%, with a sensitivity of 98.5% and a per-sample specificity of 98.8% (Fig. 6a). Full concordance metrics are shown in Table 1. For sensitizing mutations DelEx19 and L858R, 3 discordant calls made by ERASE-Seq were supported by tissue data. One DelEx 19 call was negative by ERASE-Seq but positive by ddPCR and in tissue. In conclusion, all 4 discordant calls were true positives, with sequencing proving more sensitive. For the resistance mutation T790M, 1/3 of the calls made by ERASE-Seq were supported by tissue. Because resistance mutations are expected to arise in the course of treatment, the lower tissue support is not surprising for the T790M alteration. A scatter plot of allele frequencies for the two technologies (Fig. 6b) shows concordance of AF values for sequencing versus ddPCR (Y = 1.3), with an R^2^ coefficient of 0.85.

**Table 1:** Correlation and predictive measures for our sequencing assay, assuming ddPCR results as the gold standard.

	Del Ex 19 (%)	L858R (%)	T790M (%)	All ddPCR (%)
Sensitivity (PPA)	97.1	100.0	100.0	98.5
Specificity (NPA)	98.6	99.4	98.6	98.9
PPV	94.4	93.3	85.0	91.5
NPV	99.3	100.0	100.0	99.8
Concordance	98.4	99.4	98.7	98.8

Sensitivity and specificity of sequencing versus ddPCR are also shown in Fig. 6. A majority of discordant calls are supported by tissue, making the true sequencing specificity and PPV higher than the reported values vs. ddPCR.

### Clinically-actionable mutations

**Figure 7:**
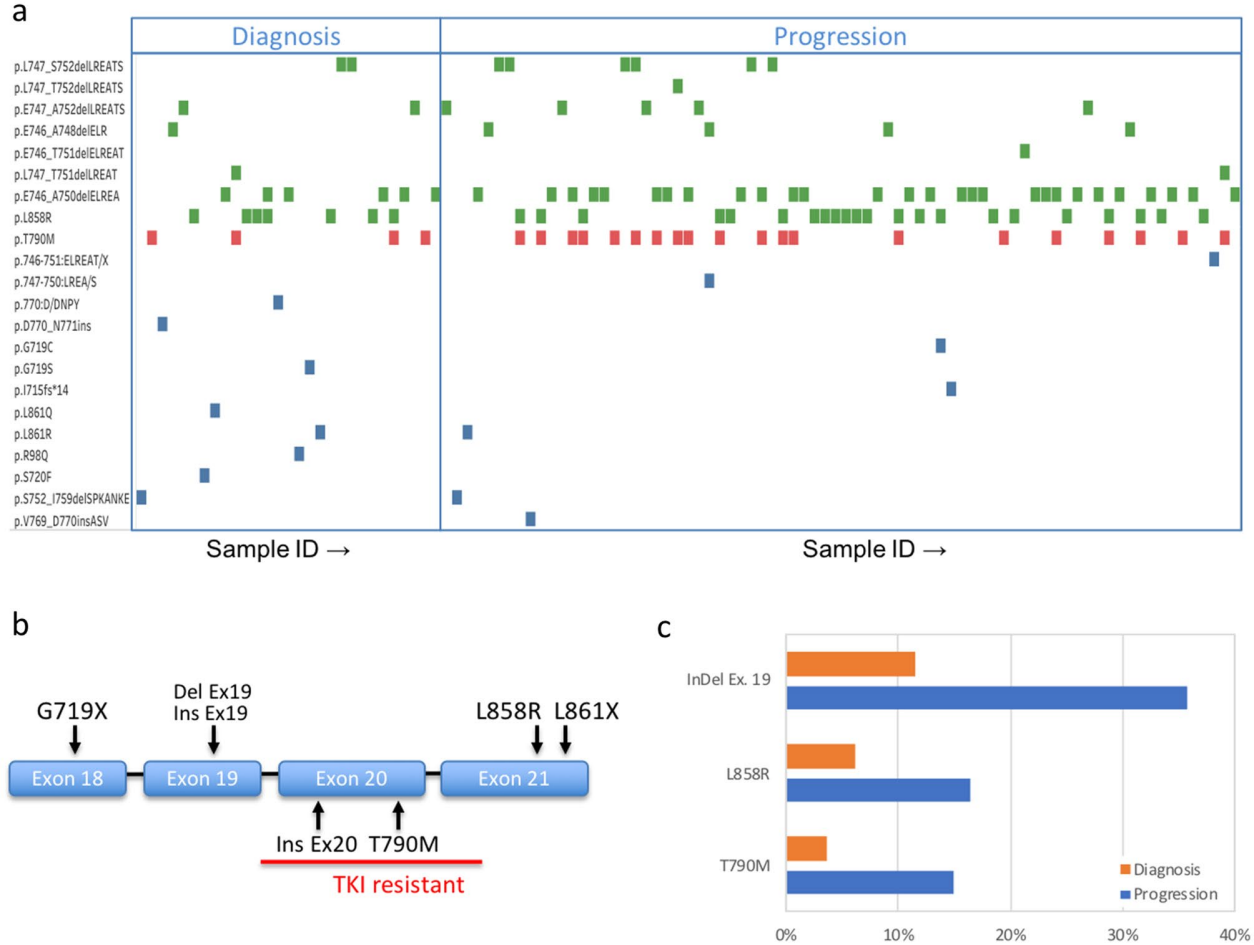
(**a**) Tyrosine kinase inhibitor (TKI) sensitivity and resistance mutations of the *EGFR* gene detected at diagnosis or at progression. Each symbol represents the detection of a specific variant (y axis) in one patient sample (x axis). Both resistance (red) and sensitivity (green) variants are detected, with all of the 6 types of TKI-relevant variants (**b**) present in the data. The prevalence of three common variant types by time point (**c**) shows the expected increase in both sensitizing and resistance mutations. Samples are not matched and represent the population of mutation-positive samples used in the study that were obtained at each timepoint.

Clinically-actionable mutations were defined as mutations with targeted therapies either approved or used as part of a clinical trial or a compassionate use basis (Supplementary Table S3)^13^. These include *EGFR* mutations governing TKI treatment sensitivity and resistance and other mutations identified in Supplementary Table S3. For each *EGFR*-positive sample, individual *EGFR* mutations are identified as a green (sensitizing) or red (resistance) marker in Fig. 7A. We detected all of the clinically-actionable alteration types previously identified in tissue across *EGFR* exons 18–21 (Fig. 7b). Prevalence of the EGFR mutations at initial diagnosis and progression was found to be within the expected range as reported in the AACR Project Genomics Evidence Neoplasia Information Exchange (GENIE) database^17^.

In addition to ddPCR-covered DelEx. 19, L858R and T790M mutations we detected G719X mutations on exon 18, insertions for both exons 19 and 20 and L861 mutation on exon 21. The prevalence of clinically-actionable *EGFR* mutations across the two sample sets at the diagnosis and progression timepoints (Fig. 7a) indicated a 3–4 × increase in the number of mutations per sample for both TKI activating and resistance mutations (Fig. 7c). Over 80% of *EGFR* resistance mutations are found to coexist with sensitizing mutations (Fig. 7a). Other mutations including *KRAS* mutations, *ALK* mutations, and *BRAF* mutation V600E are found. For all of these actionable mutations, the number of cases where an actionable variant is detected via MAPs sequencing but not ddPCR is outlined in Fig. 1.

## Discussion

### Molecular amplification pools

The use of molecular amplification pools (MAPs) and the ERASE-Seq caller are fundamentally different from other approaches to improve sensitivity and specificity in cfDNA sequencing. While UMIs attempt to track amplification reaction results for each starting molecule, MAPs are used to track amplification reaction results for pools of a few thousand starting molecules by physical separation into different reaction tubes. Because tagging of each MAP is performed post-amplification using index barcodes, this approach avoids the challenges of applying UMIs to limited-input samples.

A background-aware caller, ERASE-Seq, then utilizes data from each pool as well as a number of reference DNA pools. A statistical confidence score is considered the primary pass/fail criterion for variants (Fig. 2)^14^. In contrast, existing liquid biopsy approaches rely on UMI approaches to reduce noise^8,9,18–20^, or combine UMIs and background statistical noise reduction models^9^.

Both MAPs and ERASE-Seq are widely applicable and can be adapted for use with data from any workflow with read depths of at least 5000 × per molecular pool. This conveys the method broad applicability to virtually any cfDNA sequencing panel^14^. Single- molecule barcoding workflow limitations, including the requirement of very deep sequencing to produce consensus reads, reduced efficiency in the attachment of the molecular barcodes, and PCR errors introduced in the molecular barcodes themselves, are avoided^12^.

### Variant detection performance

Orthogonal blood-to-blood concordance testing is ideal because it removes questions of tumor heterogeneity and time-dependent variability and tests sensitivity/specificity in a real-world setting. The clinically-approved ddPCR testing was performed independently of sequencing results, as part of the standard of care. Sequencing results were generated in a blinded manner and compared to ddPCR. We obtained excellent sensitivity (98.5%) and specificity (98.9%) when comparing the MAP/ERASE-Seq approach to ddPCR results (Table 1). It should be noted that while ddPCR was considered a truth table, ERASE-Seq and ddPCR perform similarly: Of 7 discordant calls, 4 calls present in sequencing data only were concordant with tissue data, indicating that real-life PPV is 97%, versus 91.5% with respect to ddPCR (Table 1). Variant detection is likely input copy-number limited in samples containing less then 10 ng cfDNA, where only an average of 3 copies support a 0.1% AF call. High-confidence variant detection is supported to 0.1% for single base substitutions with a lower limit of detection (LOD) of 0.03% for insertions and deletions (indels). The lower indel LOD is due to the absence of an appreciable noise background for this type of alteration.

Low limits of detection are necessary in order to successfully serve a large number of patients for whom the circulating tumor DNA (ctDNA) variant frequencies are often below 0.5%^3,5,8,10,18^, a regime where recent orthogonal studies have shown that there is significant room for improvement. Recent studies evaluating results from four different UMI-based sequencing clinical test providers in challenging sample types^6,7,21^ found most discordance to be in the low AF range between 0.1 and 1%. Publications comprising late stage samples, however, report much higher blood-to-tissue and blood-to-blood agreement for UMI-based commercial tests, and robust concordance is obtained in late stage patients with higher AF variants^8,10,19,20^. For example, a recent study used ddPCR as a confirmatory assay and reported a high positive percent agreement (> 99%) above 0.3% AF, but limited ddPCR testing to sequencing-positive samples^10^.

While work remains in defining standard metrics and calculation methodologies before alternative approaches can be accurately compared, the MAPs approach demonstrated robust sensitivity / specificity in low AF region of 0.1–1% when compared to gold-standard ddPCR assays in clinical testing.

### Population-level analysis in lung cancer

This data set presents a liquid biopsy mutational profile atlas for lung cancer. Many earlier liquid biopsy validation reports have focused on closely monitoring response to treatment in a relatively small number of patients^9,18,22^, or data collated from multiple cancer types^4,8,10^. This data set has focused on a larger clinical data set in lung cancer cfDNA. In addition to the prevalence of commonly mutated genes covered by our 56 genes panel- *TP53*, *EGFR* and *KRAS* (Fig. 3), we also detected clinically-important mutations of lower prevalence.

A large dataset also enables a comparison of our liquid biopsy results to the somatic molecular signature of solid tumors with the same tissue of origin. The question asked was: is this a pan-cancer mutational profile, or is it specific to adenocarcinoma of the lung (over 80% of the patient population tested)? To that end, we mined the TCGA database to assemble similar unique mutation counts per gene for lung as well as melanoma, colon and bladder cancer. Agreement of cfDNA data with tissue lung adenocarcinoma data set (R^2^ = 0.91, Fig. 5) is significantly better with respect the same distribution for melanoma, colon and bladder cancers (Supplementary Fig. S2C,D,E; R^2^ = 0.51, 0.54, 0.86, respectively). The result strengthens the case that we are indeed detecting a lung signature.

The presence of clonal hematopoiesis (clonal hematopoiesis of indeterminate potential , or CHIP) variants has been recently identified as a possible source of false positives for cfDNA data^23,24^. To address this we propose criteria based on recently-characterized CHIP-associated mutation profile in blood cell samples^25,26^ (see Methods) and found 21% of samples to harbor mutations associated with clonal hematopoiesis (Fig. 3a). They are *DNMT3*A (28), *JAK2* (19), and KIT (5) variants previously observed in lymphomas but not lung cancers. Such variants have been found in about 10% of healthy subjects above 65 years of age, and associated with adverse outcomes^25^. They represent 11% (52/485) of the mutations detected in 21% of samples in this study. Within the *TP53* gene, 42% of variants are CHIP-associated, and 60% lung cancer-associated (COSMIC data), with a small group of variants previously observed in both. All of the clinically-relevant variants of the *EGFR*, *ALK*, *BRAF* and *KRAS* genes are unambiguously lung cancer-associated and do not meet CHIP criteria; therefore, the presence of CHIP-associated variants doesn’t affect clinical decision making.

### Clinical utility

Clinically-actionable mutations were defined as mutations with targeted therapies either approved or available for clinical trial/compassionate use (Supplementary Table S3)^13^. *EGFR* mutations governing TKI treatment sensitivity and resistance are of central importance (Fig. 7). We identified all of the clinically-actionable alteration types previously identified in tissue across *EGFR* exons 18–21 (Fig. 7). In addition to the high-prevalence variants validated via ddPCR, we also observe rare G719X and L861X sensitizing mutations as well as low-prevalence Ex19 insertions (sensitizing) and Ex20 insertions (resistance) (Fig. 7). All 7 types of clinically-actionable EGFR alterations previously identified are represented^14^ (Fig. 7b)^27^. The prevalence of clinically-actionable *EGFR* mutations across the two sample sets at the diagnosis and progression timepoints (Fig. 7a) indicated a 3–4 × increase in the number of mutations per sample for both TKI-activating and resistance mutations at progression (Fig. 7c). This is likely due to both higher tumor DNA content associated with disease progression, as well as the emergence of resistance mutations like T790M.

In addition to *EGFR* mutations, variants present in the *KRAS*, *ALK* and *BRAF* genes are actionable, providing either targeted therapy or therapies on a clinical trial basis, or an indication of resistance to therapy and are detected for a number of samples (Figs. 4, 5). Our list of actionable mutations (Supplementary Table S3) was recently shown to provide clinical response when liquid biopsy was used to guide therapy selection^13^. KRAS mutations associated with primary resistance to gefitinib and erlotinib^28^ were identified in 41 cases, and the G12C with particularly strong evidence of resistance and therapeutic interest^29^ was detected in 13/41 cases. As shown by previous studies, cfDNA sequencing provided useful information when tissue sequencing was unavailable (70% of cases) and delivered new information relating to the emergence of resistance after tissue-based targeted therapy. Of a total of 237 cases where both sequencing and ddPCR assays were used, in 55 cases (23%) sequencing provided actionable information not available from ddPCR. These were either additional *EGFR* mutations detected by NGS or other actionable genes. The results demonstrate our ability to non-invasively detect both targeted mutations and resistance mutations with high sensitivity from a majority of lung cancer patients. While excellent results have been previously obtained using molecular barcodes in advanced lung cancer populations, the validation of this alternative method points to the possibility of combining MAPs and molecular barcoding in order to further improve sensitivity for future studies.

### Limitations

While it is possible to determine CNVs from sequencing data, the metrics for CNV detection haven’t been well characterized and are omitted from this analysis. CNV detection is not a major marker for lung cancer therapy, but will be important for other indications. In addition, fusions are not targeted by the current amplicon panel. Either purpose-built amplicons^30,31^ or hybrid capture^31^ will need to be employed to detect gene fusions. Like previous studies in NSCLC, this study patient population was primarily advanced-stage patients (Stage III/IV), representative of the distribution of staging at diagnosis and first progression. The stage distribution is representative of the patient population under treatment.

In summary, we performed orthogonal clinical validation for sequencing and analysis of cfDNA samples that relies on molecular amplification pools (MAPs) and ERASE-Seq, a background-aware statistical variant caller^14^. In a 356-sample lung cancer data set, we found excellent concordance between MAP/ERASE-Seq analysis and matched blood tested via ddPCR (98.5%), indicating that sequencing correctly identifies variants present in the ctDNA fraction down to 0.1% AF in clinical samples. For a third of cases tested by both methods, sequencing uncovered additional actionable mutations with respect to ddPCR, both in the *EGFR* gene, and other relevant genes (*KRAS*, *ALK*, and *BRAF*). While ddPCR assays provide higher sensitivity for a limited number of variants, sequencing via MAPs showed improved discovery of actionable variants as it couples high sensitivity with wider coverage of clinically actionable mutations.

## Materials and methods

### Ethical considerations and patient inclusion

Patient inclusion for this study followed the CIRCAN (“CIRculating CANcer”) enrollment criteria at HCL described previously^1^. All consecutive patients with NSCLC who were routinely screened for molecular alterations over a 2-year period were eligible for inclusion in this study. The prescription of *EGFR* routine molecular screening, mandatory for advanced non-squamous NSCLC, was solely the responsibility of the treating physician. Notably, national recommendations for screening for *EGFR* mutations (both *EGFR* activating mutations and p.T790M), *ALK* rearrangements, and four emerging biomarkers (KRAS, BRAF, HER2, and PIK3CA mutations) have been available since 2010. Additionally, patients with a less advanced stage of NSCLC or patients carrying other tumor types (e. g. mixed histology, never smokers) might also have been included in this screening upon approval by their local multidisciplinary tumor board.

All samples and medical data used in the CIRCAN study were anonymized. Sample collection and processing protocols were approved by the regional ethics committee Lyon Sud Est IV (CPP L15-188 11/04/2015; amended by L16-160 09/21/2016) and French National committee in Informatics (CNIL 15-131 01.12.2015). Written informed consent for total blood sampling was obtained from all patients included in the study. All methods were carried out in accordance with relevant guidelines and regulations.

### cfDNA sample collection

Blood samples (3 × 10 mL) were collected in K2 EDTA tubes (BD, 367,525, 18 mg) within the framework of the CIRCAN routine patient management at Lyon University Hospital. As a consequence, some clinical data are missing, since the justification of the prescription is not an obligation for the clinicians. Plasma samples from NSCLC patients were collected for the detection of somatic alterations in cfDNA in the setting of routine patient management at diagnosis (in cases where FFPE tissue was unavailable or biopsy was not contributive) or during disease progression. In France, EGFR T790M mutation detection in blood samples is the preferred method for tumor resistance genotyping in this setting. Blood sampling was performed (1) at diagnosis and (2) during progression alongside of regular follow-up CT-scan (usually performed quarterly).

Extracted cfDNA was collected in one tube (60 µL), split for ddPCR, beaming and NGS assays. All extracted cfDNA was used. DNA was quantified by Qubit (Thermo Fisher Scientific). Typical cfDNA input were 0.5–80 ng (10 µL max) for the NGS assay, 0.5–63 ng (8 µL max) for the ddPCR assay, and 1–159 ng (20 µL max) for the BEAMing assay.

### Tissue data

Where available, tissue sequencing data was obtained from the treating physician. FFPE tumor samples were micro-dissected to select areas of the sample with the highest percentage of tumor cells and the smallest amount of normal tissue. Hence, samples were constituted of at least 15%-20% tumor cells. These samples were then analyzed using a customized AmpliSeq library and next-generation sequencing (PGM, Life Technologies, Carlsbad, CA, USA). Gene-level alteration presence/absence for the target mutations overlapping the ddPCR assays was reported and compared to cfDNA NGS and ddPCR data.

At diagnosis, all tumor cases were histologically or cytologically confirmed on FFPE biopsy specimens and EGFR sensitizing mutation detection was performed either on FFPE tumor samples or using cfDNA in case of tumor tissue genotyping failure as part of routine practice. RECIST measurements were performed/documented for each patients.

The tissue NGS (Thermo Fisher Scientific technology, in-house panel of oncodrivers)^1^ and ddPCR assays were performed by investigators without having any prior knowledge of clinical data; this included not having any previous results from initial mutation detection tests.

### BEAMing and droplet digital PCR cfDNA measurements

OncoBEAM is a highly sensitive and quantitative digital PCR platform utilizing Beads, Emulsion, Amplification and Magnetics (BEAMing). This platform is CE-IVD labelled and produced by Sysmex Inostics (Hamburg, Germany, EU). Detailed analytical considerations for this assay have been previously described^32^. Here, we used the OncoBEAM RAS CRC kit and OncoBEAM *EGFR* assay which enable the screening of 34 and 32 somatic genomic alterations respectively in RAS and *EGFR* genes in one run. All experiments were performed according to the supplier’s IVD recommendations for clinical application (Instructions for Use, IFU).

The sensitive and quantitative QX100 droplet digital PCR system from Bio-Rad (ddPCR, Bio-Rad, Hercules, CA, USA) combines a water–oil emulsion droplet technology with microfluidics (Bio-Rad, 186-3005). Detailed analytical considerations for this type of assay have been previously described^1,33^.

Comprehensive validation of the AF thresholds for sensitivity and specificity of the ddPCR and BEAMing tests were completed previously^1,32^ using Horizon Discovery reference standards as well as clinical samples with paired FFPE samples.

### Targeted next-generation sequencing cfDNA library preparation

cfDNA libraries were created using the multiple targeted amplicon technology provided by Swift Biosciences according to the manufacturer’s instructions (56G Oncology Panel Kit, Swift Biosciences, Ann Arbor, MI, Cat. No AL-56248). Sample loading was tuned to obtain a minimum of 5000 × read median depth per MAP. Fastq files, obtained by the demultiplexing of base-call files, were analyzed using the ERASE-Seq pipeline.

### Bioinformatics

All samples consisted of 2 MAPs, which were indexed as separate samples and sequenced at read depth above 5000 ×. The resulting fastq files generated by HCL laboratory were analyzed using the Fluxion Biosciences ERASE-Seq pipeline modified for a two-MAP data input and to maximize sensitivity/specificity for clinical use in lung cancer samples. Implementation and performance details for the general ERASE-Seq variant caller have been previously published^14^. Briefly, residual adapters and primers were trimmed using Trimmomatic^34^ and Cutadapt^35^. Cleaned reads were aligned to hg19 (GRCh37) using the MEM algorithm of BWA^36^. For indel calling, base indels were realigned using GATK^37^. LoFreq^38^ was used to identify all possible variants for the two sample MAPs and background reference sample MAPs. Custom Perl scripts were then used to parse pile-up data into a matrix containing read counts for each sample and control replicate at each panel variant, and the data matrix was then processed in R, using a negative binomial test to quantify the significance of enrichment between variant count observations in sample and control replicates^14^. ERASE-Seq uses this model to assign a confidence score (p-value) to each possible sample variant call. For each possible variant, if the multiple testing-corrected p-value is above a cutoff/threshold α (here, 0.05), then the null hypothesis cannot be rejected, suggesting no mutation present in the sample as compared to control runs. Conversely, if the null hypothesis can be rejected, i.e., *p* value < α, then a mutation call is made for the variant in question. Whereas previously negative binomial tests and p-value thresholds were used to determine the presence of significant copy differences between sample and control runs in expression data, here a similarly derived p-value threshold determines the statistical significance of mutated copies in the sample DNA MAPs with respect to control MAPs. Our preferred method using 2 MAPs and a 0.05 *p* value threshold eliminates a majority of false positive calls. Remaining FP calls are eliminated using strand bias and coverage criteria, yielding a final per-variant specificity^14^.

Standard ERASE-Seq analysis was further modified to take into account variant clinical prevalence in order to achieve excellent per-sample specificity and high sensitivity for a large set of clinically-actionable variants (Supplementary Table S4). Cancer-specific prevalence was determined using the total number of verified somatic variant observations in the Catalogue of Somatic Mutations in Cancer (COSMIC) database^39^. Ultralow calls at 0.1% AF and above were made for variants with over 100 verified somatic observations (258 SNVs); these included the 3 variants covered by the orthogonal ddPCR tests (DelEx19, L858R, and T790M). High local background for T790M mutations in our training set prompted an increase to 0.13% AF for this variant only. Calls at 0.2% AF and above were made for variants with over 5 COSMIC observations (1,186 SNVs). The remainder of approximately 100,000 variants were called if an AF above 1.0% was observed. In addition to the detection of clinically-actionable variants, this approach covered other possible variants with associated clinical value at high sensitivity. Variant lists for each category are listed in Supplementary Table S3. At each AF level, probability p(AF) that one false positive variant (FP) is called at each position is summed up to give the overall per-sample FP rate, or specificity. False positive rates for the ERASE-Seq variant caller have been measured to be < 1/300,000 variants at > 1%, 0.2/10,000 at 0.2%, and 0.8/10,000 at 0.1%^14^. Therefore, using our clinically-relevant caller, the expected FP rate for low and ultralow variants below 1% is 0.02 + 0.025 = 0.045 per sample. We therefore expect under 1 FP call over 20 samples, or a sample specificity of over 95%; this lines up with specificity results obtained with respect to ddPCR.

### Identification of hematopoietic variants

A number of recent publications have highlighted the presence of CHIP-derived variants in cfDNA samples and their possible role as false positives in liquid biopsies^23,24,40^. The gold standard for determining the origin of such variants is to sequence the white blood cell (WBC) fraction independently form the plasma^23,24,40^. While we do not have access to reference WBC sequencing information, we propose a practical methodology for correctly labeling CHIP variants in liquid biopsy data. Recently-published evidence showed that > 85% of CHIP variants have been observed in hematopoietic and lymphoid tissue samples^25,26^. We therefore classify any variant in the 0.1–20% AF range previously observed in hematopoietic and lymphoid tissue (COSMIC) as CHIP-associated. The prevalence of CHIP-derived variants is clearly identified in our data set and mirrors data found in WBC sequencing studies^25,26^.

### Sensitivity and specificity calculations

Sensitivity, specificity, concordance, and false positive rate are used to compare the performance of ERASE-Seq with several published low-frequency DNA variant detection approaches. Sensitivity, or positive percent agreement (PPA), is defined as (True Positives)/ (True Positives + False Negatives) and expresses the fraction of ddPCR positive calls made using the MAP/ERASE-Seq sequencing method. Specificity, or negative percent agreement (NPA) is defined as (True Negatives)/(True Negatives + False Positives), and expresses the ratio of samples expected to be negative by ddPCR that are called by the sequencing data. In some cases, the false positive rate is reported, defined as (False Positives)/(False Positives + True Negatives). This is alternatively expressed as false positive calls per 10,000 variant tests (False Positive Rate * 10,000). Concordance is defined as (Number of Concordant Tests)/(All Tests). These measures for our MAPs-based clinical ERASE-Seq pipeline vs. ddPCR are shown in Table 1.

A few important considerations that affect performance comparisons deserve mention. Orthogonal sequencing and ddPCR tests were administered independently of each other in our study and data from both is available for a majority of patients; this is preferable to studies where ddPCR is used as a confirmatory assay to positive calls from sequencing^10^. The ERASE-Seq analysis was first applied to a 120-sample training set and caller parameters were optimized to maximize sensitivity and specificity. The same pipeline parameters were then fixed and applied in a blinded manner to the full 356-sample set.

Sample inclusion criteria were defined as all samples meeting a minimum 5000 × per MAP read depth, and were included in the concordance analysis via ddPCR. For tissue concordance, some previous studies report overall concordance^6,7^, while others exclude samples based on the absence of ctDNA as defined by no alterations being detected^8^. This can lead to disparate conclusions from the same data set. We report both metrics when describing tissue concordance.

The assay limits of detection (LOD) are defined as the lowest variant detected and may be much lower than the high > 95% confidence allele frequency (AF) range. Comparison to ddPCR data was performed within the high-confidence AF range for that specific variant, generally above 0.1% for our sequencing assay. This matches the reported AF range for the Bio-Rad ddPCR assays used in the vast majority of tests.

## Supplementary Information

Supplementary Tables.

Supplementary Figures.

## Data Availability

The datasets generated during and/or analyzed during the current study are available from the corresponding author on reasonable request.
