# Supplementary material for: Sensitivity, specificity, and accuracy of a liquid biopsy approach utilizing molecular amplification pools
Source: Sci Rep. 2021 May 24;11:10761. doi: 10.1038/s41598-021-89592-8 (PMC8144209; doi:10.1038/s41598-021-89592-8)

Supplementary Figures

Title: Sensitivity, Specificity, and Accuracy of a Liquid Biopsy Approach Utilizing Molecular Amplification Pools

Jessica Garcia[1&], Nick Kamps-Hughes[2&], Florence Geiguer[1], Sébastien Couraud[3], Brice Sarver[2], Léa Payen[1&&], Cristian Ionescu-Zanetti[2*&&]

[1] Laboratoire de Biochimie et Biologie Moléculaire, Groupe Hospitalier Sud, Hospices Civils de Lyon, 69495 Pierre Bénite, France. ; Hospices Civils de Lyon Cancer Institute, CIRculating CANcer (CIRCAN) program, 69495 Pierre Bénite, France.

[2] Fluxion Biosciences, Alameda, California, USA

[3]Acute Respiratory Disease and Thoracic Oncology Department, CIRculating CANcer (CIRCAN) program ; Lyon Sud Hospital, Cancer Institute of Hospices Civils de Lyon, France

& - Co-first authors

&& - Co-last authors

*- Corresponding author (cristian@fluxionbio.com)

Supplementary Figure Legends

Supplementary Figure S1. Distribution of TP53 variants associated with lung cancer, CHIP (clonal hematopoiesis of indeterminate potential), and other cancers. Total associations by type of each individual variant based on COSMIC data.

Supplementary Figure S2. The number of unique mutations detected per gene is plotted for our liquid biopsy data set in lung (a) as compared to tissue-based cancer atlas data (B-D). The four solid tumor data sets analyzed in the TCGA cancer genome atlas were lung adenocarcinoma (b), melanoma (c), colon (d) and bladder cancer (e). The analysis was restricted to genomic regions covered by both tests, with the highest prevalence genes shown.

Supplementary Figure S1 TP53 variant classification by sample

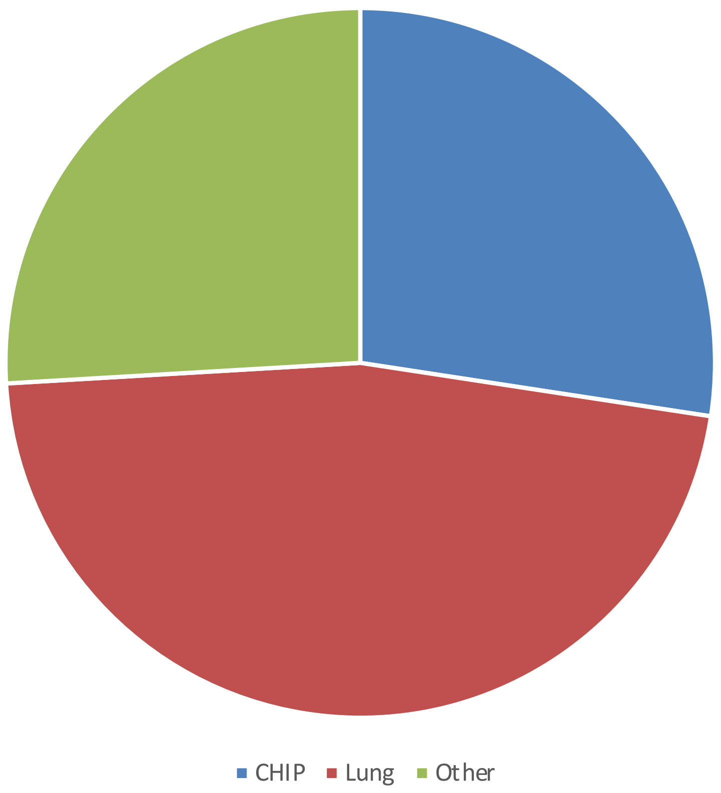

Supplementary Figure S2- Liquid biopsy variants detected relative to cancer data in TCGA

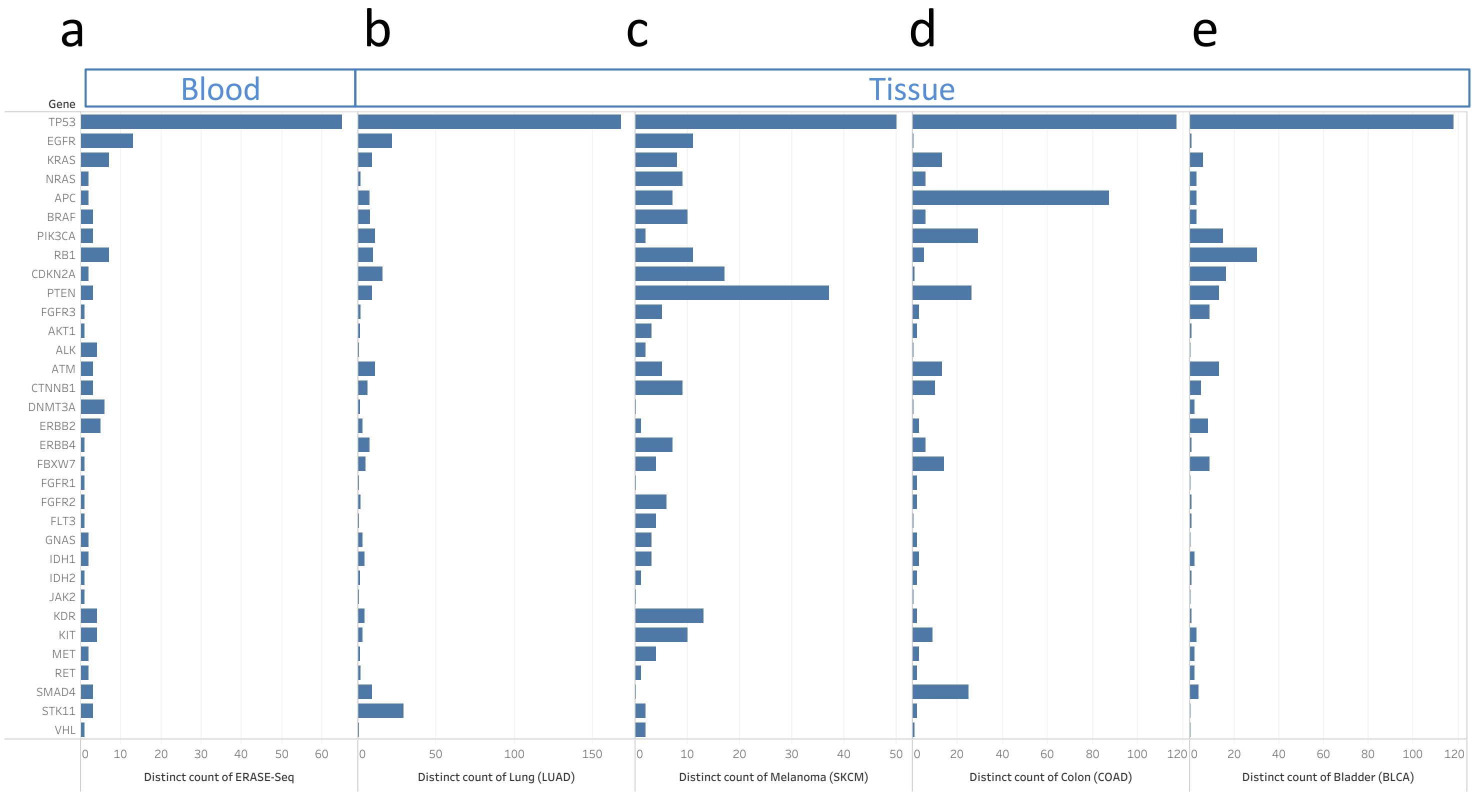

Supplement: Supplementary file 2 — Supplementary Figures. [file 41598_2021_89592_MOESM2_ESM.pdf]
